# On the makeup of high-impact reviews in ecology

**DOI:** 10.1016/j.eehl.2026.100233

**Published:** 2026-03-12

**Authors:** Stavros D. Veresoglou, Evgenios Agathokleous

**Affiliations:** a State Key Laboratory of Biocontrol, School of Ecology, Sun Yat-sen University, Shenzhen 518107, China; b Climate and Atmosphere Research Center, The Cyprus Institute, 20 Konstantinou Kavafi Street, Nicosia 2121, Cyprus; c Research Center for Global Changes and Ecosystem Carbon Sequestration & Mitigation, School of Ecology and Applied Meteorology, Nanjing University of Information Science & Technology, Nanjing 210044, China

**Keywords:** Computerized text analysis, Ecology and Evolution, Narrative arc, Psychometrics, Scientific writing

## Abstract

To maximize the impact of our contributions, we strive to perfect our scientific writing. Much of the existing guidance on how to effectively structure reviews originates from anecdotal opinions and guidelines set out by the journals themselves. This makes it less clear what ultimately determines the number of citations of review papers. Citation frequencies partly depend on the topic of the review, and on the innovative nature of the ideas within the review. However, the language norms and the narrative flow within a review might also play an important role in the eventual acceptance of the ideas. Here, we analyzed the text of review papers published in 2020 in the top ten journals in ecology. Citation counts correlated with two of the four psychometrics tested, as well as the word count of the contributions, explaining an aggregate of 1.9% of total variation. We further observed relationships in citation counts with two descriptors of the article structure. We identify linguistic traits correlated with citation frequency in ecology, with potential relevance across other disciplines. A solid theoretical background on best practices in review writing would be transformative in terms of contributing to tools for further improving the impact of reviews, but also to assist their preliminary editorial evaluation.

## Highlights

• Computerized text analysis opens up opportunities to improve writing.
• We explained over 1.9% of citation frequency in reviews on ecology from 2020.
• Clout and authenticity explain subsequent citation frequency in reviews.
• A “U” type staging process and early cognitive tension contribute to citation success.
• We contribute a solid theoretical background on psychometrics in ecology.

## 1 Introduction

Honing scientific writing skills and enhancing communication efficiency are top priorities in academia [1,2]. Despite attempts to increase the outreach of papers through measures such as plain-language summaries, editorial comments, and blogs [3], the language of scientific articles is becoming less accessible to the general public [[4], [5], [6]]. Science is increasingly dealing with more specialized topics. This fact could at the same time be a warning sign that the scientific community does not appreciate what aspects of scientific writing resonate with the readers of those articles. Many guidelines for scientific writing are anecdotal [[7], [8], [9], [10]], although this situation has improved recently with computerized text analysis methods [[11], [12], [13]].

Ecology can be distinguished from most other science, technology, engineering, and mathematics disciplines: the high stochasticity of the systems on which ecology works makes the findings less conclusive than, for example, those in chemistry and mathematics [14]. Some ecologists even go as far as to classify ecology as a “soft” science [15] (i.e., less rigorously quantifiable than sciences such as physics or chemistry). The less conclusive nature of the articles in ecology implies that, for most research topics, multiple competing articles with comparable results exist. In such “high competition” scenarios, scientific writing is more likely to determine the citation frequency of an article than in other disciplines. For example, longer manuscripts in ecology tend to be cited more often [16], and there is a considerable boost in citations for publishing open access [17]. More authors and the inclusion of native English speakers can also raise citation frequency [17,18].

### 1.1 Objectives and hypotheses

We (i.e., the scientific community) underappreciate how language norms determine citation counts. Even though primary literature contributions can be quite technical, this is not the case with reviews in ecology, for which we anticipated that, to a certain degree, academic success reflects other language traits. Here, we quantify success in the form of citation counts, despite being a proxy with many apparent shortcomings [19]. In particular, we hypothesized (hypothesis 1) that there are strong correlations between the citation frequency of reviews from top journals in ecology and the four summary psychometric statistics, authenticity, analytic ability, clout, and tone, which are assessed with Linguistic Inquiry and Word Count (LIWC-22; [20]), a computerized program for text analysis.

Another element of scientific writing is the structure of papers. A major constituent of the structure is the division of the text into appropriate sections, such as the introduction, discussion, and conclusion, but it further captures the preferential use of words to hone the contents of each section and facilitate the narrative [20]. For example, early on in a paper and at the end of it, we expect the authors to establish and reiterate the basic background of the paper by introducing places, hypotheses, and ideas, respectively. To do so, they must disproportionately use prepositions and articles, which we refer to as “staging” [20,21]. We might expect, as a result, that staging in heavily-cited papers peaks in the beginning and the end of a paper, thus having a “U” shape, which is our second hypothesis (hypothesis 2; in Text S1, we rationalize what we mean by a U shape in descriptors of the Narrative Arc). The optimal narrative structure, however, may be context-dependent, and our U-shaped hypothesis, derived from general language studies, is one of several possible models (e.g., see Ref. [22] for an alternative, hump-shaped structure). Similarly, we might expect that the use of cognitive processing words such as “think”, “understand”, and “believe” in the manuscript would peak in the second half of the main text of the paper [20]. We might expect, as a result and because of the crude resolution of narrative arc analyses, to observe that the cognitive tension in heavily-cited reviews monotonically increases throughout the contribution more than in less heavily cited reviews (hypothesis 3).

## 2 Methods

### 2.1 Sources of data

On April 22, 2025, we retrieved from the Web of Science (WoS) all papers published in 2020 in the following ten journals (alphabetically): *Annual Review of Ecology Evolution and Systematics* (*Annu. Rev. Ecol. Evol. Syst.*), *Current Opinion in Insect Science* (*Curr. Opin. Insect Sci.*), *Ecosystem Services* (*Ecosyst. Serv.*), *Ecography*, *Ecology Letters* (*Ecol. Lett.*), *Frontiers in Ecology and the Environment* (*Front. Ecol. Environ.*), *Global Change Biology* (*Glob. Change Biol.*), *Journal of Ecology* (*J. Ecol.*), *Nature Ecology and Evolution* (*Nat. Ecol. Evol.*), and *Trends in Ecology and Evolution* (*Trends Ecol. Evol.*). We targeted a single year because papers get cited more over time, making it tricky to compare papers from different years, with results generalizable across years. The journals were selected from the *Journal Citation Reports 2023*—the most recent edition available at the time of the search—as the top ten journals with the highest impact factor in the “Ecology” category that each published at least ten review articles. We specifically addressed review articles because the primary literature is often quite technical, which could complicate language analysis. The applied review criterion was the filtering option of WoS for review contributions, which reflects relatively low stringency. We thus did not discriminate between systematic and narrative reviews—a limitation of our study. Book reviews were excluded. A total of 333 review articles met our inclusion criteria. For each article, we extracted metadata (e.g., title, authors, journal) and citation counts as recorded in WoS up to the search date. The text of each review was manually copied into a text file. Figure and table captions, acknowledgment sections, boxes, and author information were removed: these elements may obscure the textual analysis yet contribute to the citation count [23], but do not adhere well with the general writing norms.

### 2.2 Computerized text analysis

We used LIWC-22 [20] to assess the language norms and narrative arcs of the reviews. LIWC provides four summary statistics on the language. These variables are continuous, scaled from 1 to 100. Analytical thinking (Analytic) captures the degree to which people use words that suggest formal, logical, and hierarchical thinking patterns [24]. Authenticity reflects the degree to which a person self-monitors, such as in conversations between close friends [25]. Clout reflects the writing norms that people with high social status tend to use [26]. Sentimental tone (Tone) captures the relative use of negative (values below 50) over positive (values above 50) words in the text [27]. LIWC-22 uses closed-vocabulary approaches, meaning that the dictionary is predefined and has been previously used for extensive analyses, which contribute insights into interpreting the output. For the narrative arc, texts were divided into five segments, and the LIWC-22 independently scored each segment for staging and cognitive tension, as briefly explained earlier [11]. Staging tracks the use of language used to establish the basic background of the paper by introducing places, hypotheses and ideas. Cognitive tension monmitors the use of cognitive processing words such as “think”, “understand” and “believe”. We focused on the raw output rather than the summary statistics of LIWC-22. Even though the default dictionary of LIWC has not been developed specifically for academic text, and thus the interpretations of absolute psychometric scores might be misleading, we mainly relied on comparative statistics across papers. We interpret LIWC metrics as descriptive linguistic markers derived from a general corpus.

### 2.3 Hypothesis 1: Strong correlations between citation frequency and psychometric statistics

**Fig. 1:**
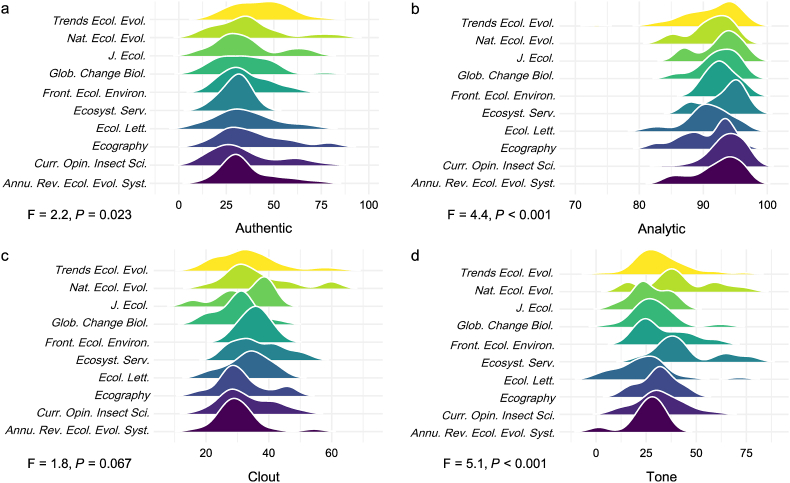
Psychometric statistics (a: authenticity, b: analytic ability; c: clout; d: sentimental tone) for the 333 review articles included in our analysis, captured as ridgeline plots. The statistics at the bottom left of each panel assess the degree to which we observe differences in the psychometric indices across the journals. Note that all four psychometric indices are assessed on a scale from 1 to 100, and there are pronounced differences in their variation across review articles; i.e., most review articles have an analytic ability above 80, whereas their authenticity varies throughout the range of the respective statistic. *Annu. Rev. Ecol. Evol. Syst*, *Annual Review of Ecology Evolution and Systematics*; *Curr. Opin. Insect Sci.*, *Current Opinion in Insect Science*; *Ecosyst. Serv.*, *Ecosystem Services*; *Ecol. Lett., Ecology Letters*; *Front. Ecol. Environ.*, *Frontiers in Ecology and the Environment*; *Glob. Change Biol.*, *Global Change Biology*; *J. Ecol.*, *Journal of Ecology*; *Nat. Ecol. Evol.*, *Nature Ecology and Evolution*; Trends Ecol. Evol., *Trends in Ecology and Evolution.*

Our preliminary analysis (Fig. 1) revealed large (three out of the four tests were significant with a median F value of 3.3) differences across the journals. Combining citation statistics into a single linear model could miss important sources of variation. We adopted a meta-analytical approach instead. For each of the psychometric statistics, we assessed the correlation coefficient with the citation count, square-root transformed, per journal. We used standard random-effects meta-analytical approaches to process those statistics (Text S1).

### 2.4 Hypotheses 2 and 3: The use of an appropriate narrative contributes to citations

We divided the text into five segments and assessed the Narrative Arc statistics on LIWC-22. To assess the narrative arc of staging and cognitive tension, we fitted two linear models: a first-order linear model and a quadratic model (for the quadratic model, the segments contained an additional predictor with the following values per segment: 1, 4, 9, 16, 25). We selected the model with the smallest Akaike Information Criterion value. We classified the values into the four categories according to the sign of the quadratic (quadratic model: a positive value manifested a “U” shape, whereas a negative value manifested a humped shape) or linear (linear model: a positive value manifested a monotonic increase, whereas a negative value manifested a monotonic decrease) term. For our analysis, we used the subset of reviews that were classified in the first (Q1: 25% most cited reviews per journal) and fourth (Q4: 25% least cited reviews per journal), which constituted the binary response variable (Q1: 1; Q4: 0). The two predictors we considered were journal identity (categorical) and classification type (one of “U”-type, humped, monotonic increase, or monotonic decrease). We carried out the analysis with and without *Curr. Opin. Insect Sci.* observations because of the unusually small size of the main text of those papers (Fig. S1).

## 3 Results

### 3.1 Summary of linguistic metrics

The median authenticity was 33.7 and varied among journals (F = 2.2, *P* = 0.023; Fig. 1a), ranging from 29.6 (*Curr. Opin. Insect Sci.*) to 41.7 (*Trends Ecol. Evol.*). In other words, a value of 30 (e.g., *Curr. Opin. Insect Sci.*) indicates that the journal publications rank lower than 70% of texts in the LIWC reference database, ranking third in terms of perceived authenticity.

Median analytic thinking was 93.05 with differences across journals (F = 4.4, *P* < 0.001; Fig. 1b), ranging from 90.7 (*Ecol. Lett.*) to 94.5 (*Ecosyst. Serv.*). Based on the LIWC-22 framework [20], an analytic thinking score of 91−95 indicates an extremely formal, logical, and abstract writing style that prioritizes precision over narrative flow. As previously mentioned about the issue of increasingly specialized writing, these results indicate a need to lower the analytic scores (e.g., 60−80) to aim for broader audiences and greater narrative flow, such as by incorporating active voice, pronouns, and examples.

The median clout was 31.9 and differed among journals (F = 1.8, *P* = 0.067; Fig. 1c). With a cautious, humble, and collaborative tone that avoids strong claims or assertions, a clout value of 32 indicates low perceived authority, confidence, or social influence in the text [20]. Common contexts of such writing include interdisciplinary studies, qualitative and exploratory research, texts emphasizing limitations or uncertainty, and early-career academic writing.

The median sentimental tone was 30.1, with significant variation among journals (F = 2.2, *P* = 0.023; Fig. 1d), ranging from 23.3 (*Ecol. Lett.*) to 40.2 (*Ecosyst. Serv.*). According to the LIWC-22 framework [20], a sentimental tone of 23−40 indicates considerably low emotional expression, where the text is neutral, detached, or subtly negative in affective tone.

### 3.2 Hypothesis 1: Strong correlations between citation frequency and psychometric statistics

**Fig. 2:**
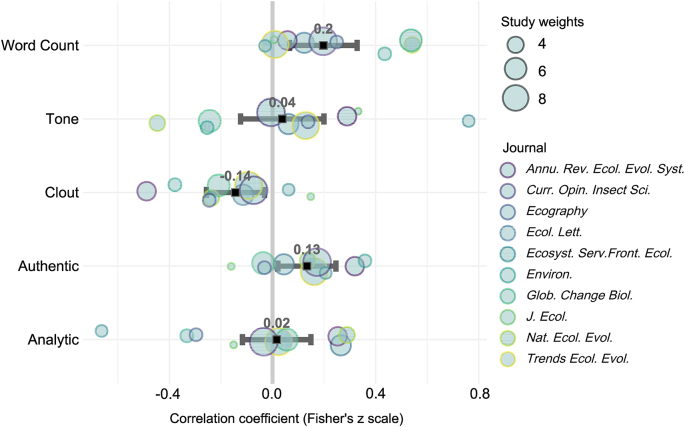
Meta-analytical results (i.e., method of moments with a DerSimonian-Laird estimator) for the correlation coefficient (Fisher’s z-scale transformed) of the four psychometric statistics, as well as the word count with the square root of citation counts. The results are presented in the form of a forest plot, and the error bars represent 95% confidence intervals. Word count and authenticity showed positive correlations with citations, whereas clout exhibited a negative correlation.

There were nonzero correlation (Fisher z-scale transformed) coefficients for authenticity (Q_B_ = 5.4, *P* = 0.02; Q_H_ = 3.7, *P* = 0.93), clout (Q_B_ = 6.3, *P* = 0.012; Q_H_ = 5.2, *P* = 0.81), and word count (Q_B_ = 8.74, *P* = 0.003; Q_H_ = 10.7, *P* = 0.29) (Fig. 2). Q_B_ quantifies between-group differences, whereas Q_H_ reflects model heterogeneity (Text S1). The respective back-transformed *r*^2^ estimates were 4.2‰ for authenticity, 4.88‰ for clout, and 9.93‰ for word count, accounting for a total of 1.9% of the total variance in citation frequency. Heterogeneity biases were examined with funnel plots (Fig. S1). While the total variance explained by linguistic style alone is modest (about 2%), its consistent detectability in our large-scale analysis is notable. This indicates that linguistic style represents a small but statistically independent signal within the multifactorial system of citation behavior, alongside major drivers like topic choice and author prominence. We also tried but failed to observe the relationship between citation frequency and the number of authors (Q_T_ = 2.21, ES = 0.13, *P* = 0.14; Q_H_ = 18.8, *P* = 0.027) in our dataset. However, analyzing relationships involving author count would likely require a larger sample of articles, though not a full-text analysis of each one.

### 3.3 Hypotheses 2 and 3: The use of an appropriate narrative contributes to citations

**Fig. 3:**
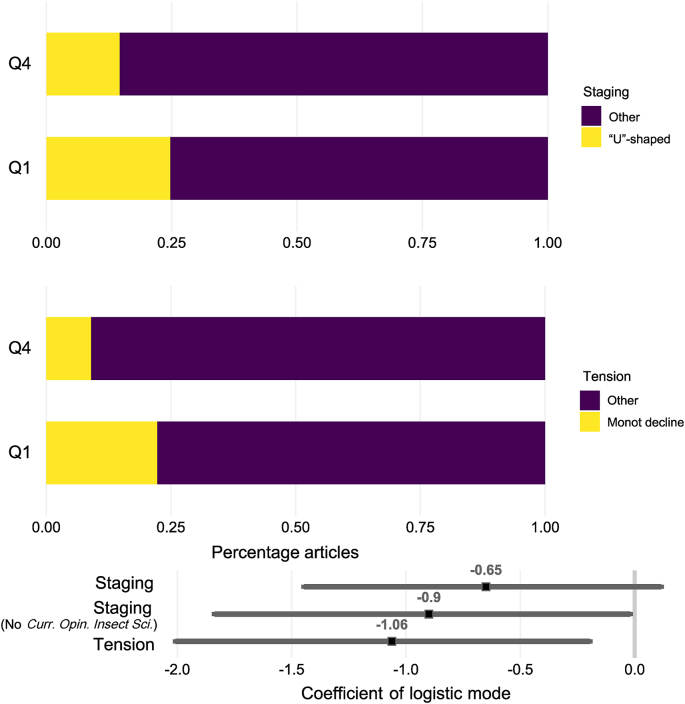
Distribution of least cited (Q4) and most cited (Q1) articles per journal in relation to (*x*-axis) the frequency of a “U”-shaped staging (top) and monotonically declining cognitive tension (bottom). We modeled the relationships via logistic regression and presented the coefficient data at the bottom of the graph.

Besides the language norms, citation frequency could also capture how well a paper is structured, which we here assessed in the form of three Narrative Arc statistics. We conducted analysis both including and excluding *Curr. Opin. Insect Sci.*, a journal with an unusually low word count (i.e., almost 800 words less than the second lowest journal; Fig. S1). Low word counts could reduce the reliability of the accuracy of the narrative Arc Analysis. The frequency of “U”-shaped staging was higher in Q1 than in Q4 papers only after excluding *Curr. Opin. Insect Sci.* (F = 4.6, *P* = 0.032); with the journal included, the statistics were not significant (F = 2.95, *P* = 0.086; Fig. 3). There was no evidence that monotonic increases in cognitive tension were more frequent in Q1 papers (F = 0.14, *P* = 0.70). In contrast, there was a greater frequency of monotonically declining cognitive tension in Q1 than in Q4 papers (F = 7.2, *P* = 0.007)*,* though the difference in frequency was not significant when *Curr. Opin. Insect Sci.* was excluded (F = 3.1, *P* = 0.076; Fig. 3).

## 4 Discussion

We present evidence that the language norms and narrative arc of reviews in ecology contribute to the citation frequency of review articles, notwithstanding well-appreciated contributors, such as the choice of topic, the innovativeness of the contribution, the authoritative nature of the contributors, and open access publishing (in agreement with hypothesis 1). We acknowledge that the aggregate of 1.9% of the total variance in citation frequency is small from a strict methodological standpoint. However, we consider it notable because we did not integrate considerations such as topic choice, quality of the ideas, and dissemination practices, which are known to greatly affect citation frequency. Thus, the detectable effect of linguistic style, while modest, adds independently to these major drivers. The statistics varied considerably with journal (Fig. 2), but there were no obvious patterns as a function of how specialized the journals were (e.g., *J. Ecol.* vs *Nat. Ecol. Evol.*) or the frequency with which they engaged in open access publishing. Language norms, however, are largely dictated by journals, and we observed considerable differences in psychometrics across journals, which limits the authors’ stylistic flexibility when they write their contributions. Moreover, many journals contribute extensive professional editing services to the papers they publish, which, to a certain degree, might homogenize their language norms. This might imply that our figures considerably underestimate the potential to further improve the citation statistics of reviews, which might have required the authors to work with the editorial teams of the journals.

The second consideration is that, in our analysis, the journals that maintained the highest impact factors did not necessarily use better writing norms than their counterparts with a lower impact factor. There are two readings on this. First, we focused on the most influential journals in the field of ecology, and all the contributions met the state-of-the-art standards for scientific writing. The second reading is that based on the relationships we observe between clout and authenticity, there remains plenty of unrealized potential for the authors to further improve their scientific writing. This is something the journals might want to consider in the future: reviewing the authors’ diagnostic checks in the form of informed feedback on how to maximize legibility. At the same time, the authors might want to engage in computerized text analysis methods by themselves to assess how well they hit home.

There was partial evidence that “U”-shaped staging (in agreement with hypothesis 2) and monotonically declining cognitive stress (in disagreement with hypothesis 3) contribute to citation frequency. Because of the categorical nature of the responses, we could not quantify the variance that could be explained with our narrative arc analyses, but there were substantial differences in the frequencies with which we observed those patterns among Q1 and Q4 reviews (Fig. 3). The “U”-shaped staging is in agreement with expectations in the literature [11]. This was not the case, however, for our observation of cognitive stress. Academic papers are quite distinct from other types of written literature in that the authors usually have to formulate sets of hypotheses early on and then factually assess the degree to which they are supported. Formulating hypotheses may require the authors to speculate, which builds cognitive stress (i.e., through speculative terms). The use of speculative language is therefore more effective at the beginning of reviews than at the end.

Academic publishing currently undergoes a major breakthrough through the gradual integration of large language models into scientific documents [28,29]. Large language models offer many opportunities to facilitate the writing process [30] but come with the downside of relying on language norms that have been used in their training material. Large language models, unless specifically trained for this purpose, could thus propagate suboptimal writing practices in the literature and prevent us from taking advantage of tools such as those we describe here. A possibility is now open to further improve artificial intelligence through integrating existing psychometric inventories [31], which makes it timely as ever to identify such psychometric relationships the way we do here.

For feasibility reasons, we focused our analysis here on ecological journals. How generalizable might our results be for journals in other disciplines? There are apparent large differences in the writing of scholarly articles across disciplines [32,33]. In addition, we observe several general trends over time, such as the increasing complexity and consistency of scientific language across disciplines [33]. There is a good chance that our observations here are generalizable across diverse disciplines that tend to use complex language. However, this is a point that requires further exploration, and our claim here can serve as a testable hypothesis that will be evaluated across contexts and disciplines.

## 5 Conclusion

We present a set of analyses supporting the idea that language norms contribute to the citation frequency of review (and possibly primary) papers in ecology. However, without dedicated manipulation experiments, there is always the possibility that we misdiagnose the culprits. For example, native English speakers may preferentially utilize specific language norms, which we identify as being cited more frequently, but these specific norms contribute little to actual citation counts. Notwithstanding this point, we present, to the best of our knowledge, the most conclusive to date analysis on how the language of scientific writing determines citations of contributions in academia. With computerized text analysis gaining popularity, it should be possible for authors to self-assess the psychometrics of their manuscripts and optimize them for a higher citation frequency.

## CRediT authorship contribution statement

**Stavros D. Veresoglou:** Writing – review & editing, Writing – original draft, Investigation, Formal analysis, Data curation, Conceptualization. **Evgenios Agathokleous:** Writing – review & editing, Writing – original draft.

## Declaration of generative AI and AI-assisted technologies in the manuscript preparation process

We used no AI tools in writing the present contribution.

## Declaration of competing interest

The authors declare that they have no known competing financial interests or personal relationships that could have appeared to influence the work reported in this paper.
