# Supplementary material for: On the makeup of high-impact reviews in ecology
Source: Eco Environ Health. 2026 Mar 12;5(2):100233. doi: 10.1016/j.eehl.2026.100233 (PMC13087775; doi:10.1016/j.eehl.2026.100233)
Supplement: Multimedia component 1 [file mmc1.docx]

Supplementary Information

Contents

Table S1. Statistics on quantitative articles

Fig. S1. Funnel plots

Fig. S2. Citation trajectories for articles published in 2005

Fig. S3. Citations (*y*-axis) relationships with the psychometrics clout and authenticity (*x*-axis) for two journal articles

Text S1: Detailed materials and methods

Text S2: Adherence by the PRISMA guidelines

Text S3: Detailed output for the four psychometrics

Table S1. Statistics on quantitative (“yes”) and non-quantitative (“no”) articles in our dataset. We only explored further Ecology Letters and Global Change Biology that presented more balanced sets of contributions. Quantitative articles comprise amongst others meta-analytical syntheses or studies that involved the collection and analysis of primary data.

|  | **yes** | **no** |
| --- | --- | --- |
| **ANNUAL REVIEW OF ECOLOGY, EVOLUTION, AND SYSTEMATICS** | 0 | 25 |
| **CURRENT OPINION IN INSECT SCIENCE** | 0 | 87 |
| **ECOGRAPHY** | 0 | 13 |
| **ECOLOGY LETTERS** | 12 | 20 |
| **ECOSYSTEM SERVICES** | 5 | 7 |
| **FRONTIERS IN ECOLOGY AND THE ENVIRONMENT** | 3 | 10 |
| **GLOBAL CHANGE BIOLOGY** | 8 | 32 |
| **JOURNAL OF ECOLOGY** | 2 | 8 |
| **NATURE ECOLOGY & EVOLUTION** | 1 | 17 |
| **TRENDS IN ECOLOGY & EVOLUTION** | 3 | 87 |


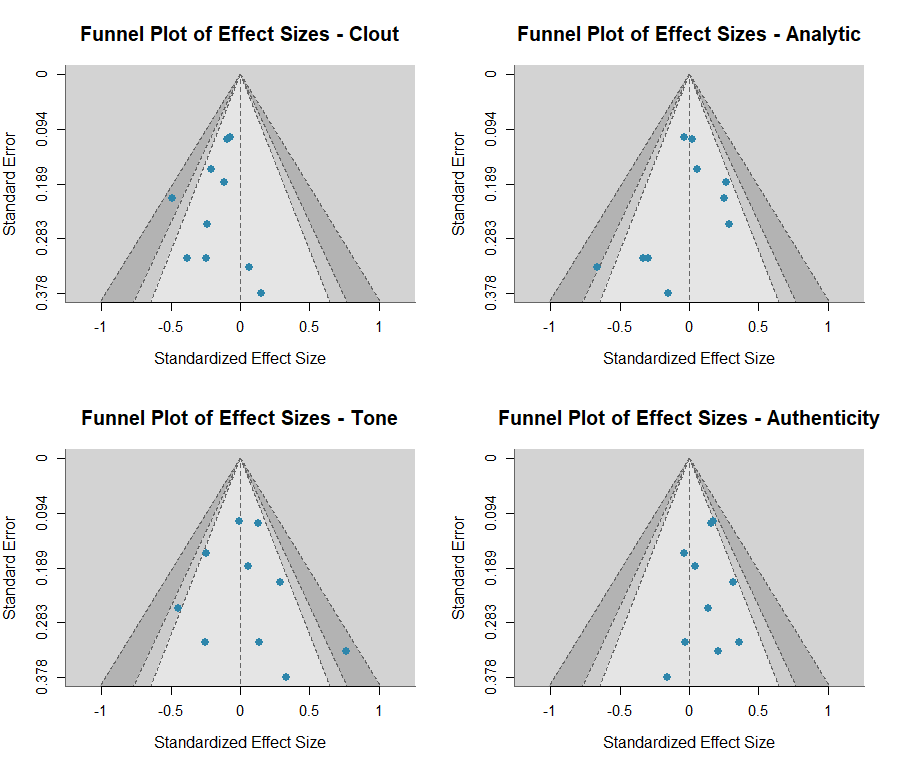


Fig. S1. Funnel plots for the four psychometrics. We did not anticipate any publication bias but we prepared the plots to adhere by the PRISMA guidelines. The three shades of grey in the funnel plots correspond to 90%, 95% and 99% CIs. The take home message is that the possibility of having a publication bias in our analyses was highly unlikely. Each point in the plot corresponds to one journal title (*n* = 10).


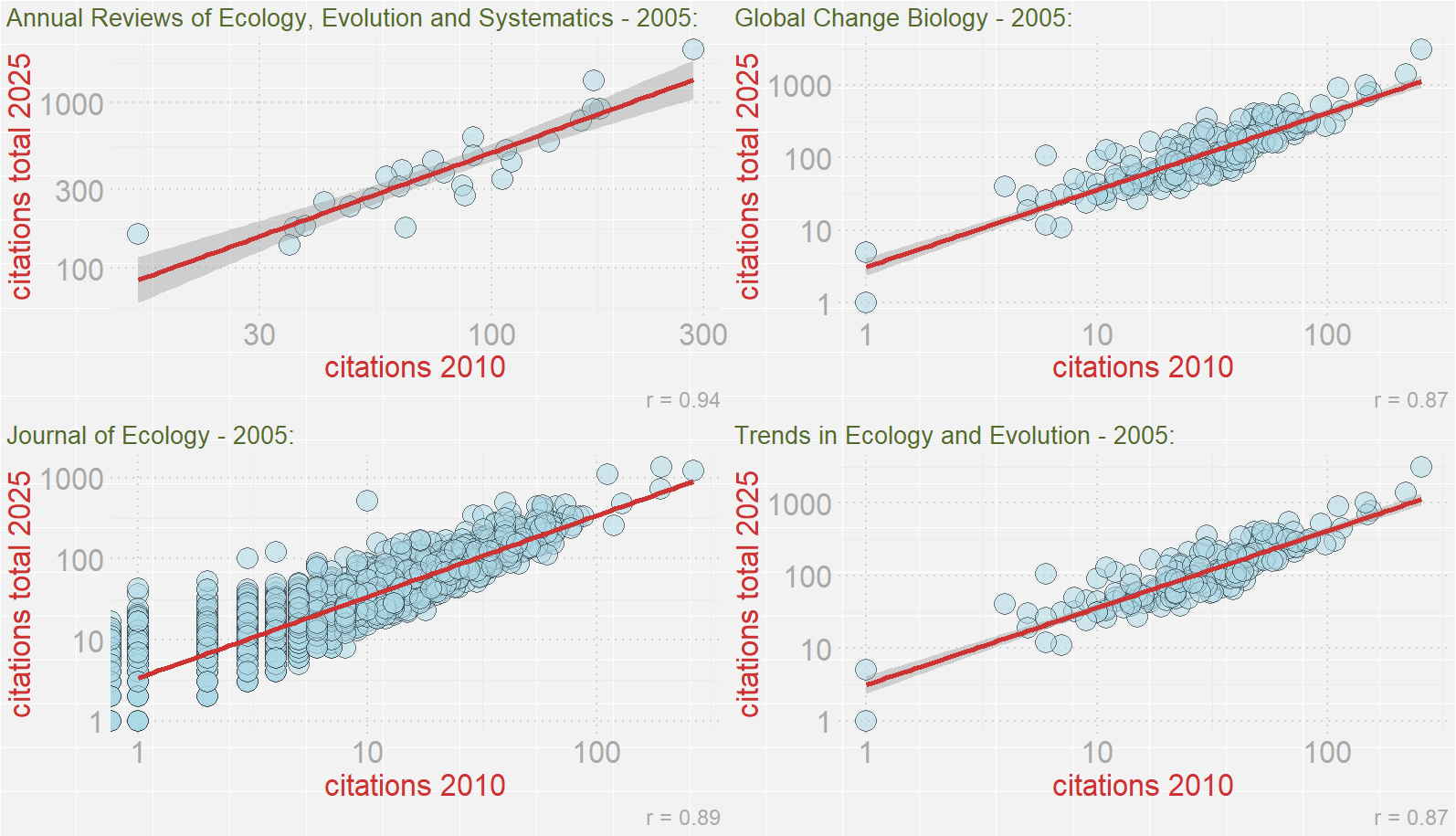


Fig. S2. Citation trajectories for articles published in 2005 in four journals, evaluated in the end of 2010 (i.e. five years after) – in the *x* axis and on the 7^th^ of November 2025 (*y*-axis). We report on the correlation coefficient in each case. The take home message is that citation trajectories five year after publication change little subsequently in the citation history of an article.


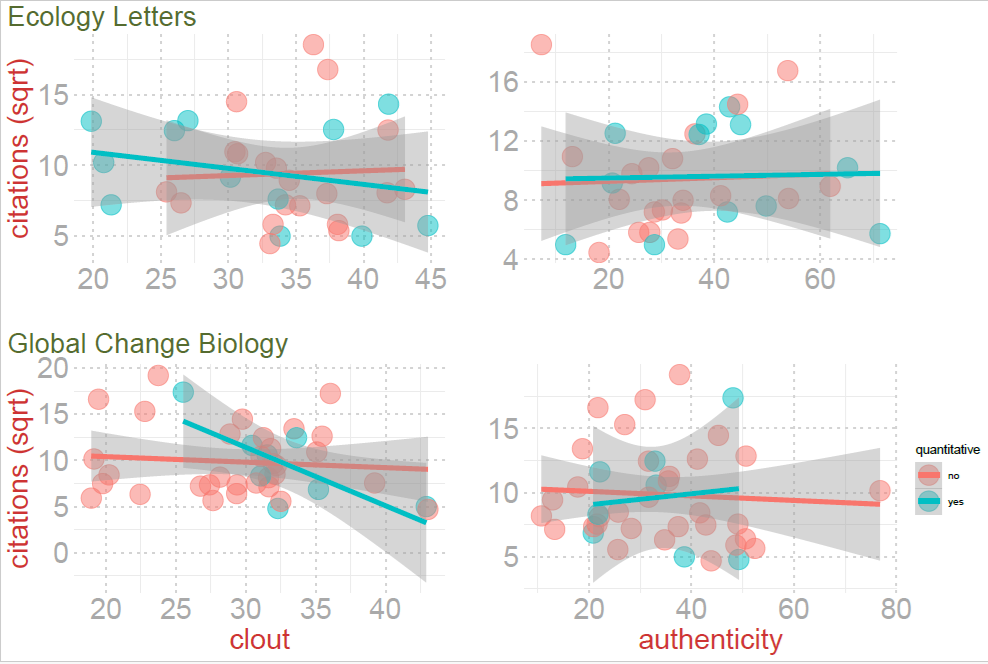


Fig. S3. Citations (*y*-axis) relationships with the psychometrics clout and authenticity (*x*-axis) for two journal articles. We discriminate here between quantitative and non-quantitative contributions and observe small differences between the two groups of contributions.

Text S1: Detailed materials and methods

*Limitation of the 'Review' Classification:* Our analysis relies on the Web of Science 'Review' filter, which encompasses a broad spectrum of article types, from narrative syntheses to systematic reviews and meta-analyses (but see Fig. S3). These genres have different methodological constraints (e.g., PRISMA guidelines) and purposes, which likely influence both linguistic style and citation patterns. Our findings should therefore be interpreted as broad correlates across this heterogeneous category, and future work should investigate how narrative effects differ between specific review types.

*Comparative journal statistics*: We fitted general linear models with square-root-transformed citation counts as a response variable and journal name as a categorical predictor.

*Meta-analyses*: We extracted correlation coefficients between the square transformed citation counts and the following five covariates: (a) word count; (b-e) the four psychometrics. Correlation coefficients were transformed to a Fisher’s z-scale and journal weights were proportional to the number of observations per journal (σ^2^ ≈ 1 / n-3). The meta-analysis was based on the method of moments and we used a random effects DerSimonian-Laird estimator with the R library *metafor*. We report on both the model statistics (Q_B_) and the heterogeneity statistics (Q_B_) in the main manuscript, but we present the full output in the form of Text S3. There was no evidence for a publication bias (Fig. S1) and we could not find support on whether the classification of reviews into quantitative reviews or qualitative ones explained any additional variation (Fig. S3). We further report on how our meta-analyses adhered by the PRISMA guidelines in Text S2.

*Narrative Arc*: LIWC gives narrativity scores in relation to three different features: staging, plot progression, and cognitive tension: the text is divided into five equal segments and each of them is assessed independently (raw scores) in relation to the respective features (Boyd, et al., 2020). The use of five segments is the default option of LIWC and the programmers advice against changing that parameter. Scoring is in relation to the aspects of the language that give rise to the feature: e.g. for staging this should be the frequency of prepositions and articles, whereas in the case of cognitive tensions this should be cognitive process words such as “think”, “believe”, “understand” and “score”. These scores are subsequently z-score transformed.

Our approach involved assessing whether a quadratic term improved the fit of a first order linear model and, in the case it did, the sign of that quadratic term, or if it did not the sign of the linear term. This way we arrived at four different possibilities of progression “U” or “humped” in the case of quadratic models performing better, monotonically increasing or decreasing in the case that first order linear models performed better. We subsequently extracted the top 25% performing in relation to citations articles per journal (Q1) and the bottom performing (Q4). We used quartiles as a means of homogenizing citation trajectories across diverse outlets and chose the two extreme quantiles to avoid the complications of fitting models with ordinal predictor variables. We combined the two types of articles into a binomial way: 1: top performing; 0: bottom performing, and fitted generalized linear logistic models with the specific binary variable as response and the predictors journal and the questioned type of progression as predictors.

Boyd, R. L., Blackburn, K. G., & Pennebaker, J. W. (2020). The narrative arc: Revealing core narrative structures through text analysis.

Text S2: Adherence by the PRISMA guidelines

(We only report here on the way our analysis meets twelve of the key consideration criteria in the PRISMA guidelines. We actually used a modelling approach, and this is reflected on the way we handled some of the considerations)

*1 Present the details of the search*

We report on the database we used and we used all eligible articles in our search.

*2 Study inclusion and exclusion criteria*

We report on the three inclusion criteria (i.e. year of publication, journals and contribution type) we used for the studies in our database.

*3 PRISMA Flow Diagram*

We used all eligible articles and there was no need for a PRISMA flow diagram.

*4 Data Extraction Process*

We specify that we manually extracted the text from each of those articles and how we processed it.

*5 Weighting effect sizes*

We specify that we used the inverse of variance for the z-score transformed correlation coefficients

*6 Specifying meta-analytical model*

We specify that we used a random effects model with a DerSimonian-Laird estimator

*7 Dealing of heterogeneity*

We report on heterogeneity statistics within the main manuscript but also in Text S3

*8 Assessment of publication bias*

We present in Fig. S1 funnel plots

*9 Non-independence among study outcomes*

The journals were the units of the analysis. We report how we handled them.

*10 Specify the software used*

We do so and specify that we used R..

*11References*

We make all data available, including the references

*12 Data used for the meta-analysis*

We make all data available, including the references

Text S3: Detailed output for the four psychometrics

> myrma<-rma(zclout, variance, method="DL", data=meta.table)

There were 50 or more warnings (use warnings() to see the first 50)

> myrma

Random-Effects Model (k = 10; tau^2 estimator: DL)

tau^2 (estimated amount of total heterogeneity): 0 (SE = 0.0171)

tau (square root of estimated tau^2 value): 0

I^2 (total heterogeneity / total variability): 0.00%

H^2 (total variability / sampling variability): 1.00

Test for Heterogeneity:

Q(df = 9) = 5.1821, p-val = 0.8182

Model Results:

estimate se zval pval ci.lb ci.ub

-0.1438 0.0574 -2.5023 0.0123 -0.2564 -0.0312 *

---

Signif. codes: 0 ‘***’ 0.001 ‘**’ 0.01 ‘*’ 0.05 ‘.’ 0.1 ‘ ’ 1

> myrma<-rma(zanalytic, variance, method="DL", data=meta.table)

> myrma

Random-Effects Model (k = 10; tau^2 estimator: DL)

tau^2 (estimated amount of total heterogeneity): 0.0079 (SE = 0.0211)

tau (square root of estimated tau^2 value): 0.0889

I^2 (total heterogeneity / total variability): 17.89%

H^2 (total variability / sampling variability): 1.22

Test for Heterogeneity:

Q(df = 9) = 10.9605, p-val = 0.2784

Model Results:

estimate se zval pval ci.lb ci.ub

0.0163 0.0678 0.2406 0.8099 -0.1166 0.1492

---

Signif. codes: 0 ‘***’ 0.001 ‘**’ 0.01 ‘*’ 0.05 ‘.’ 0.1 ‘ ’ 1

> myrma<-rma(ztone, variance, method="DL", data=meta.table)

> myrma

Random-Effects Model (k = 10; tau^2 estimator: DL)

tau^2 (estimated amount of total heterogeneity): 0.0240 (SE = 0.0301)

tau (square root of estimated tau^2 value): 0.1550

I^2 (total heterogeneity / total variability): 39.83%

H^2 (total variability / sampling variability): 1.66

Test for Heterogeneity:

Q(df = 9) = 14.9575, p-val = 0.0921

Model Results:

estimate se zval pval ci.lb ci.ub

0.0379 0.0825 0.4588 0.6464 -0.1239 0.1996

---

Signif. codes: 0 ‘***’ 0.001 ‘**’ 0.01 ‘*’ 0.05 ‘.’ 0.1 ‘ ’ 1

> myrma<-rma(zauthentic, variance, method="DL", data=meta.table)

> myrma

Random-Effects Model (k = 10; tau^2 estimator: DL)

tau^2 (estimated amount of total heterogeneity): 0 (SE = 0.0171)

tau (square root of estimated tau^2 value): 0

I^2 (total heterogeneity / total variability): 0.00%

H^2 (total variability / sampling variability): 1.00

Test for Heterogeneity:

Q(df = 9) = 3.6820, p-val = 0.9311

Model Results:

estimate se zval pval ci.lb ci.ub

0.1337 0.0574 2.3271 0.0200 0.0211 0.2463 *

---

Signif. codes: 0 ‘***’ 0.001 ‘**’ 0.01 ‘*’ 0.05 ‘.’ 0.1 ‘ ’ 1

> myrma<-rma(zwc, variance, method="DL", data=meta.table)

> myrma

Random-Effects Model (k = 10; tau^2 estimator: DL)

tau^2 (estimated amount of total heterogeneity): 0.0070 (SE = 0.0206)

tau (square root of estimated tau^2 value): 0.0837

I^2 (total heterogeneity / total variability): 16.17%

H^2 (total variability / sampling variability): 1.19

Test for Heterogeneity:

Q(df = 9) = 10.7366, p-val = 0.2942

Model Results:

estimate se zval pval ci.lb ci.ub

0.1974 0.0668 2.9555 0.0031 0.0665 0.3284 **

---

Signif. codes: 0 ‘***’ 0.001 ‘**’ 0.01 ‘*’ 0.05 ‘.’ 0.1 ‘ ’ 1

###Narrative Arc

execute<-function(file, cola, colb)

### Different statistics on the Narrative Arc are stored at different columns - here cola:colb - 5 columns total

{

extract<-cbind(file[, cola:colb]) ### a five column data frame

comb<-apply(extract, 1, function(x) AIC(lm(x~c(1:5)), lm(x~c(1:5)+c(1, 4, 9, 16, 25))))

### we fit models with (1:5 & 1,2,4,9,16,25) or without quadratic terms

comb<-unlist(lapply(comb, function(x) which(x$AIC==min(x$AIC)))) ### we determine optimal model

coef<-cbind(apply(extract, 1, function(x) summary(lm(x~c(1:5)))[[4]][2]),

apply(extract, 1, function(x) summary(lm(x~c(1:5)+c(1, 4, 9, 16, 25)))[[4]][3]))

### based on the coefficient (i.e. quadratic positive or negative or if not linear) determine the type of curve

results<-rep(NA, length(comb))

for(i in 1:length(results)) if(comb[i]==2) {results[i]<-as.numeric(coef[i,2]<0)+1

}else results[i]<-as.numeric(coef[i,1]<0)+3

return(results) ### we return a value from 1 - 4 with the type of fit

}

### With all journals

### Group is Q1 vs Q4 articles in relation to citations within the journal

### Journal is categorical variable with the journal name

tension2<-(tension==4)

Analysis of Deviance Table

Model: binomial, link: logit

Response: tension2

Terms added sequentially (first to last)

Df Deviance Resid. Df Resid. Dev F Pr(>F)

NULL 178 158.59

journal 9 12.5579 169 146.03 1.3953 0.183652

group 1 7.2164 168 138.82 7.2164 0.007224 **

---

Signif. codes: 0 ‘***’ 0.001 ‘**’ 0.01 ‘*’ 0.05 ‘.’ 0.1 ‘ ’ 1

Warning message:

In anova.glm(glm(tension2 ~ journal + group, family = "binomial"), :

using F test with a 'binomial' family is inappropriate

#### Without Current Opinion

> anova(glm(staging2[journal!="CurrOpinInsectSci"]~journal[journal!="CurrOpinInsectSci"]+factor(group)[journal!="CurrOpinInsectSci"], family="binomial"), test="F")

Analysis of Deviance Table

Model: binomial, link: logit

Response: staging2[journal != "CurrOpinInsectSci"]

Terms added sequentially (first to last)

Df Deviance Resid. Df Resid. Dev F Pr(>F)

NULL 131 136.42

journal[journal != "CurrOpinInsectSci"] 8 6.050 123 130.37 0.7562 0.6416

factor(group)[journal != "CurrOpinInsectSci"] 1 4.604 122 125.77 4.6040 0.0319 *

---

Signif. codes: 0 ‘***’ 0.001 ‘**’ 0.01 ‘*’ 0.05 ‘.’ 0.1 ‘ ’ 1

Warning message:

In anova.glm(glm(staging2[journal != "CurrOpinInsectSci"] ~ journal[journal != :

using F test with a 'binomial' family is inappropriate
